# Long-term stabilization of hydrogen peroxide by poly(vinyl alcohol) on paper-based analytical devices

**DOI:** 10.1038/s41598-019-49393-6

**Published:** 2019-09-10

**Authors:** Tuchpongpuch Boonpoempoon, Wanida Wonsawat, Takashi Kaneta

**Affiliations:** 1 Department of Chemistry, Faculty of Science and Technology, Suan Sunandha Rajabhat University, Bangkok, Thailand; 2 Department of Chemistry, Graduate School of Natural Science and Technology, Okayama University, Okayama, Japan

**Keywords:** Microfluidics, Lab-on-a-chip, Sensors

## Abstract

Stabilizing reagents that can be deposited onto paper is an important issue for researchers who depend on paper-based analytical devices (PADs), because long-term stability of the devices is essential in point-of-care testing. Here, we found that poly(vinyl alcohol) (PVA) would stabilize hydrogen peroxide placed on a paper substrate following exposure to air. Horseradish peroxidase was employed as a sample in colorimetric measurements of PADs after hydrogen peroxide and 3,3′,5,5′-tetramethylbenzidine were deposited as substrates in an enzymatic reaction. The addition of PVA to hydrogen peroxide significantly suppressed its degradation. Concentrations of PVA that ranged from 0.5 to 2%, increased the duration of the stability of hydrogen peroxide, and the results for a PVA concentration of 1% approximated those of 2% PVA. Storage of the PADs at 4 °C in a refrigerator extended the stability of the hydrogen peroxide containing 2% PVA by as much as 30 days. The stability of hydrogen peroxide without PVA was degraded after one day under room temperature.

## Introduction

Portability and ease of operation are the qualities that allow miniaturized analytical devices to satisfy the requirements of chemical analyses outside sophisticated laboratories. Over the past decade, paper-based analytical devices (PADs) have been used to achieve point-of-care testing. PADs were first reported in 2007 by the Whitesides’ group who demonstrated paper bioassay determinations of glucose and protein that were inexpensive, low-volume and portable^1^. Since that first report, the use of PADs has grown rapidly, as described in many review articles^2–7^.

The most popular detection scheme of PADs has been colorimetry, which quantifies an analyte by color intensity of the product produced in a chemical reaction with reagents deposited on a paper substrate. Selective and specific quantification of analytes is accomplished by several chemical reactions that include complex formation between metal ions and chelate reagents^8–10^, aggregation of nanoparticles^11–14^ and enzymatic reactions^15–18^. In the colorimetric measurements, the reagents involved in the chemical reactions are deposited and dried in a specified zone of the PADs. Technicians simply add a sample into a channel or a zone to react an analyte with deposited reagents.

An important issue for PADs is the development of simple detection schemes that promote instrument-free detection. In general, colorimetry can be achieved by taking images of the PADs, which is followed by image processing to measure the color intensity or hue^19–21^. Obviously, judgment of the concentrations using only the naked eye would be an attractive option in terms of portability and ease of operation. Several groups have reported instrument-free detection using distance-readout^22–28^, time-readout^29–31^ and counting of the zones colored by a chemical reaction^32–34^. These detection schemes are promising for analyses outside of a laboratory, which is often required in developing countries and in poorly equipped laboratories.

Another issue to be resolved is the stabilization of reagents on the paper substrate, which amounts to the stability of the PADs themselves during storage. When we need chemical analyses out of the laboratory, the PADs must be stable during transport and storage. Despite the recognition of its importance by researchers, only a few studies have focused on the stability of enzymes^35–37^ and antibodies^38^ on paper substrates. The stabilization of horseradish peroxidase (HRP) was achieved by trehalose and SU-8 epoxy novolac resin^35^ whereas poly(vinyl alcohol) (PVA) has also been used as a reagent for stabilization of enzymes in the PADs^39–41^. However, to the best of our knowledge, no other successful stabilization of chemical reagents in PADs has yet been achieved, which is unfortunate.

In this study, we stabilized hydrogen peroxide deposited on a paper substrate using PVA. Hydrogen peroxide is a useful reagent in enzyme assays^42,43^ and chemiluminescence measurements^44,45^. The use of hydrogen peroxide, however, requires either a fresh solution or the preparation of PADs just prior to measurement. Ramachandran *et al*. employed sodium percarbonate as a source of hydrogen peroxide for ELISA in point-of-care devices. However, a fresh solution of sodium percarbonate was added in the study^46^. This implies that sodium percarbonate would also be unstable when deposited onto a paper substrate. In this study, we found that PVA can stabilize hydrogen peroxide deposited onto a paper substrate for at least 30 days with storage in a refrigerator whereas the deposited hydrogen peroxide immediately degraded without PVA at room temperature. The stability of hydrogen peroxide was evaluated in a reaction of HRP and 3, 3′, 5, 5′-tetramethylbenzidine as enzyme and substrate, respectively, using PADs prepared with different concentrations of PVA and stored under different temperatures.

## Experimental Section

Materials. All chemicals were of analytical reagent grade. Reagent solutions were prepared using deionized water (18.2 MΩ·cm) purified using a Milli-Q System from Merck Millipore (Millipore Co. Ltd., Molsheim, France). Horseradish peroxidase (HRP), 3, 3′, 5, 5′-tetramethylbenzidine (TMB) and poly(ethylene oxide) (average molecular weight, 100,000) were purchased from Sigma-Aldrich (St. Louis, MO, USA). Bovine serum albumin (BSA), PVA ([-CH(OH)CH_2_-]_n_, n = 1,500–1,800), sodium dihydrogen phosphate and disodium hydrogen phosphate were purchased from Wako Pure Chemical Industries (Osaka, Japan). Poly(vinyl pyrrolidone) (molecular weight, 1,000,000) was purchased from Polysciences, Inc. (Warrington, PA, USA). Polyethylene glycol #6,000 (average molecular weight, 7,800–9,000) and two different PVAs with n = 500 and n = 2,000 were obtained from Nacalai Tesque (Kyoto, Japan). The PVAs with n = 500, 1,500–1,800 and 2,000 were assigned as P500, P1650 and P2000, respectively. Hydrogen peroxide (H_2_O_2_) was acquired from Kanto Chemical (Tokyo, Japan). Preparation of a solution of TMB involved dissolving it in ethanol with the addition of 0.1 mL of 0.1 M hydrochloric acid.

Fabrication of PADs. The PADs had reaction wells arranged in 7 mm-diameter circles that were designed using Microsoft Office Power Point 2013. The PADs were printed on a sheet of filter paper (200 × 200 mm, Chromatography Paper 1CHR, WhatmanTM, GE Healthcare Lifesciences, United Kingdom) using a wax printer (ColorQube 8580 N, Xerox, CT), which was followed by heating at 120 °C for 2 min in a drying machine (ONW-300S, AS ONE Corp., Osaka, Japan). Reagent solutions were added to each well in the following order after drying: 5 µL of 35.7 mM TMB, 5 µL of 1.0 mg mL^−1^ BSA (to block the adsorption of HRP onto paper)^44^, 5 µL of 1.0 M phosphate buffer (pH 6.5) and 5 µL of 0.01% H_2_O_2_ solution without or with PVA (10 µL of 1% H_2_O_2_ was added to 1 mL of a PVA solution with 0.5, 1.0 or 2.0 w/v%). The PADs were stored in opaque boxes with moisture absorbers (silica gel) after drying completely (Supplementary Information, Fig. S1). Then, 5 µL of a 1 mg mL^−1^ HRP solution was employed as a sample. The images of the PADs were captured by a scanner (CanoScan LiDE 500 F, Canon, Tokyo, Japan) and processed using Image J software according to a procedure established in the literature^8^.

## Results and Discussion

**Figure 1:**
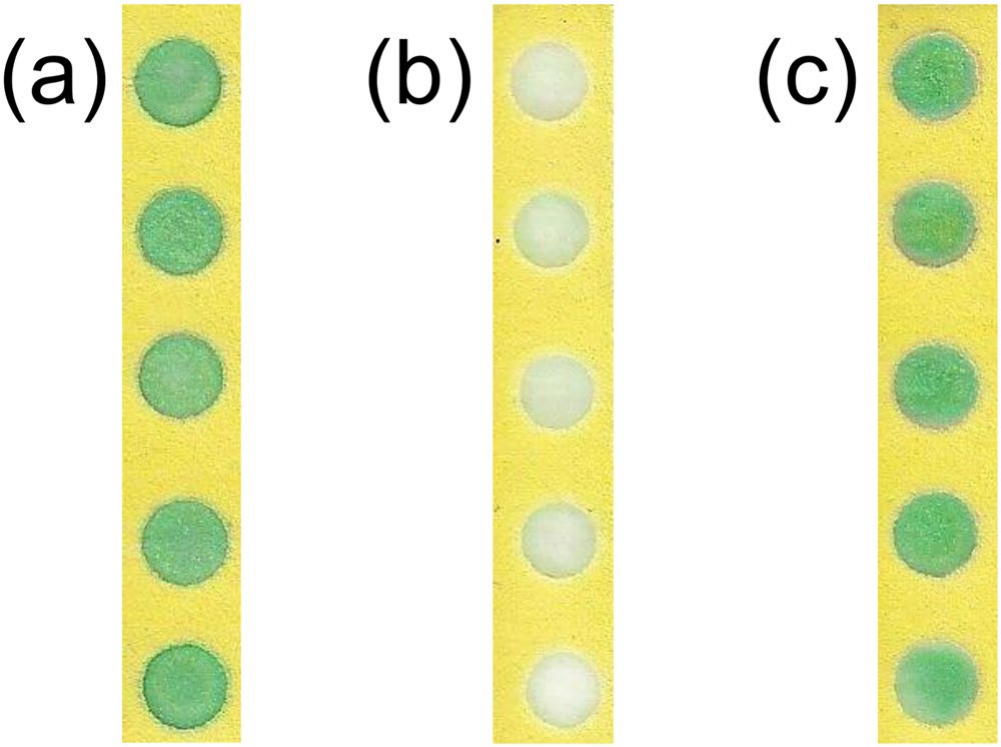
Stability of the enzyme substrates. (**a**) Freshly prepared PAD containing TMB and H_2_O_2_, (**b**) PAD prepared without TMB and stored one day at room temperature, (**c**) PAD prepared without H_2_O_2_ and stored one day at room temperature. In (**a**), HRP was added. In (**b**), TMB and HRP were added successively, and in (**c**) H_2_O_2_ and HRP were added successively.

TMB is known to be a good colorimetric reagent that changes to a blue color in the presence of H_2_O_2_ and HRP^47^. When all the reagent solutions, TMB, BSA, phosphate buffer and H_2_O_2_, were deposited in the wells of the PADs and stored at room temperature, the color intensity had reduced after one day. We assumed this could have been caused by degradation of the TMB and/or the H_2_O_2_, so we prepared two types of PADs, one without TMB and another without H_2_O_2_. After storage at room temperature for one day, the TMB and H_2_O_2_ were added to different reaction wells just before the introduction of HRP. Figure 1(a) shows the immediate results using a freshly prepared PAD containing both TMB and H_2_O_2_. The PAD was prepared by adding TMB, BSA, phosphate buffer and H_2_O_2_, and then HRP was added immediately after the PAD had dried completely. The PADs, on which H_2_O_2_ was pre-deposited with the reagents, except for TMB, were stored for one day at room temperature and exhibited significantly lower levels of color intensity, which indicated a degradation of the H_2_O_2_ (Fig. 1(b)). No degradation of the TMB appears in Fig. 1(c) where the PAD was prepared by pre-depositing the reagents, and H_2_O_2_ was added with HRP after one day. Here the PAD shows the same color intensity as that displayed in Fig. 1(a), which suggests the H_2_O_2_ had rapidly decomposed during storage at room temperature.

The degradation of H_2_O_2_ would be attributed to OH^−^ generated from water adsorbed on the paper because OH^−^ catalyzes the decomposition of H_2_O_2_, although the catalytic reaction described in the literature was performed in an alkaline medium^48^. Therefore, to confirm the degradation of H_2_O_2_ by OH^−^ at a neutral pH, H_2_O_2_ solutions were prepared with 1 mM borate buffer (pH 9) and 1 mM phosphate buffer (pH 7) and stored at room temperature. The PADs without H_2_O_2_ were prepared by adding TMB, 1 M phosphate buffer (pH 6.5) and BSA. The color intensities were 98.7 ± 1.3 for pH 7 and 69.5 ± 3.5% for pH 9 when adding the H_2_O_2_ solution and HRP to the PADs immediately after drying. Therefore, H_2_O_2_ is decomposed rapidly in a solution of at least pH 9. After storing the H_2_O_2_ solutions for one day at room temperature, the color intensities for pH 7 and 9 exhibited 55.1 ± 0.8% and 28.7 ± 0.8%, respectively (Supplementary Information, Fig. S2). The results obviously suggested that H_2_O_2_ is decomposed even at pH 7 in an aqueous solution.

**Figure 2:**
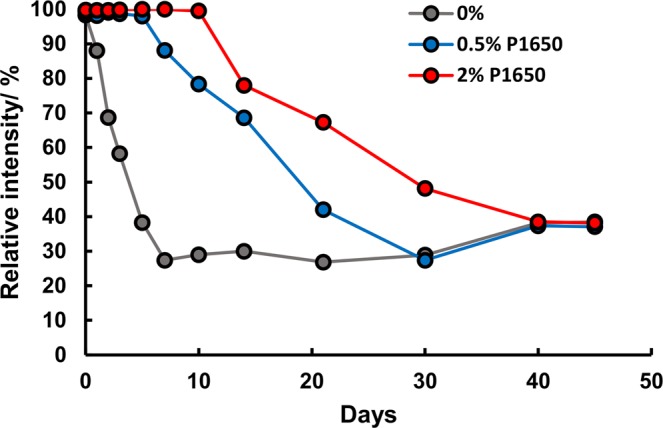
Effect of PVA concentrations on the stability of H_2_O_2_. The PADs were stored at room temperature (20 °C) for 45 days. PVA, P1650. The standard deviations for all data were smaller than the size of the marker in the figure.

To prevent the degradation of H_2_O_2_ during the storage of the PADs, we employed PVA to prevent the exposure of H_2_O_2_ to air, water in particular, because dry PVA has excellent barrier properties against permanent gases^49,50^. Fig. 2 shows the stability of PADs with the addition of different concentrations of PVA (P1650). The relative color intensities are expressed as the ratios of the one-day results to those obtained using a freshly prepared PAD. It should be noted that the standard deviations for all data were less than 1.3%, and smaller, than the size of the marker in the figure. Without PVA, the color intensity was immediately decreased daily and fell to less than 30%. Conversely, the color intensity was constant for 10 days with the addition of 2% PVA to H_2_O_2_. The stability depended on whether the concentration of PVA was 0.5 or 2% while no difference was detected for concentrations between 1 and 2% with storage at room temperature. These results obviously indicate that PVA suppresses the degradation of H_2_O_2_.

**Figure 3:**
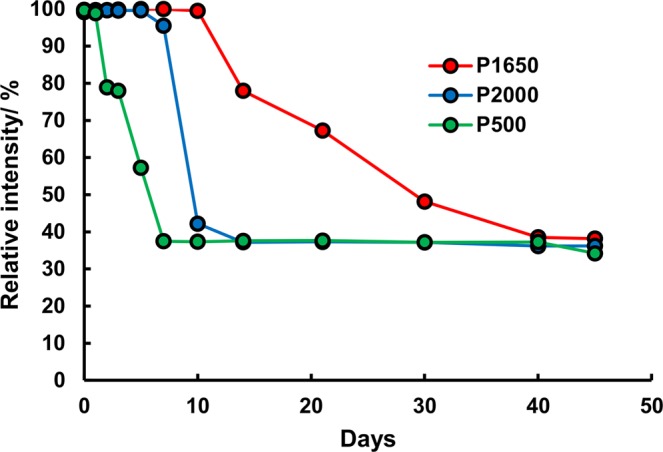
Stability of H_2_O_2_ with different PVAs. The PADs with PVA were stored at 25 °C for 45 days. The standard deviations for all data were smaller than the size of the marker in the figure.

Three types of PVAs with different degrees of polymerization were examined for the stabilization of H_2_O_2_, as shown in Fig. 3. The PVA with n = 2,000 (P2000) shows that the stabilization of H_2_O_2_ was comparable to that with n = 1,500–1,800 (P1650) whereas the PVA with n = 500 (P500) deteriorated H_2_O_2_ in two days. A feasible reason for the poor stabilization effect of P500 would be the existence of carboxyl groups in the molecule because carboxylate may catalyze the decomposition of H_2_O_2_ as well as that of OH^−^. This assumption is supported by the fact that the infrared spectrum of P500 showed an extremely intensive peak of acetate groups at 1732 cm^−1^ compared with those of P1650 and P2000 (Supplementary Information, Fig. S3)^51^. Furthermore, to clarify the effect of carboxylate on the decomposition of H_2_O_2_, the pH of the phosphate buffer solutions was adjusted to 6.5 after adding 10 mM or 100 mM sodium acetate, and these were added to the PADs. The results are shown in the Supplementary Information (Fig. S4). The color intensity for both the PADs containing 10 mM and 100 mM sodium acetate gradually reduced daily whereas the phosphate buffer without sodium acetate showed no decrease in the color intensity for four days. Therefore, it is reasonable to attribute the early degradation of P500 to the carboxylate groups. Conversely, the influence of the chain length would be insignificant because P1650 exhibited stability that was longer than that of P2000. Stability of P2000 that was slightly lower than that of P1650 was attributed to reasons other than the content of carboxylate and the chain length. It should be noted that the supplier of P1650 was different from that of P500 and P2000. This implies that P1650 could have functional groups and/or a conformation that is different from that of P500 and P2000 although this remains unclear.

**Figure 4:**
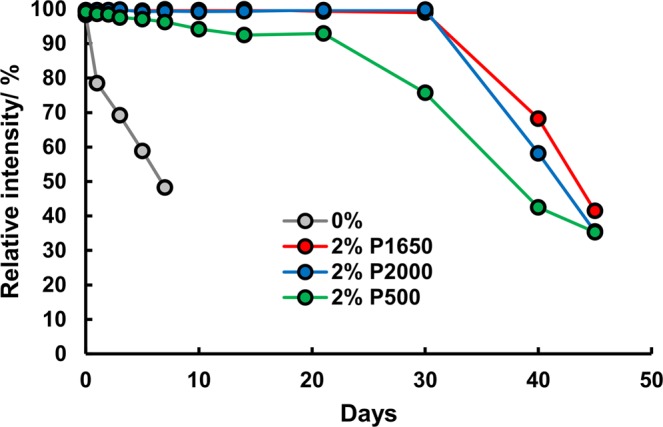
Stabilization of H_2_O_2_ on PADs stored at low temperature. The PADs without and with PVA were stored at 4 °C in a refrigerator for 7 days and 45 days, respectively. The standard deviations for all data were smaller than the size of the marker in the figure.

Ten days, however, is a short period for the storage of PADs in general use. Therefore, we attempted to extend the period of stability via storage at 4 °C in a refrigerator. As the results in Fig. 3 demonstrate, H_2_O_2_ remained stable for 30 days at a low temperature in the presence of P1650 and P2000 although the PADs without PVA and with P500 were degraded rapidly and gradually, respectively, even at 4 °C. There was no difference between a 1 and 2% concentration of PVA (P1650), and it would be difficult to further increase the concentration of PVA due to high viscosity of the solution. As shown in Fig. 4, stability was significantly improved via the addition of PVA even at a concentration as low as 2% with storage at a low temperature.

For the PADs prepared with 2% PVA, the standard deviations of intra-day measurements ranged from 0.2 to 1.1% during 45 days of storage (n = 5 for each day). The inter-day precision during 30 days was 0.6% with excellent reproducibility. Therefore, PVA proved to be an efficient stabilizer of H_2_O_2_ for deposition on a paper substrate.

We also investigated the effect of Fe(III) ion, which is known to catalyze the decomposition of H_2_O_2_. The PADs were prepared by adding all the reagents followed by 5 µL of 100 µM FeCl_3_ and were stored in a refrigerator. As expected, Fe(III) ions played the role of catalyst for the decomposition of H_2_O_2_. Surprisingly, PVA inhibited the catalytic reaction in the presence of 100 µM Fe(III) (99.9%) whereas the color intensity was decreased to 17.9% without PVA (Supplementary Information, Fig. S5). This implies that PVA prevents the contact of H_2_O_2_ with Fe(III).

It is known that several polymers prevent gas permeation as well as PVA, but among them only PVA is hydrophilic^52^. Conversely, some hydrophilic polymers possibly suppress the decomposition of H_2_O_2_ if they prevent H_2_O_2_ from contacting OH^−^ and H_2_O. Therefore, we examined three hydrophilic polymers, poly(ethylene oxide), poly(ethylene glycol) and poly(vinyl pyrrolidone), as candidates of substituent chemicals for PVA. Interestingly, these polymers also suppressed the degradation of H_2_O_2_ as well. The color intensities were 99.6% for poly(ethylene oxide) and 98.9% for poly(ethylene glycol) after 10 days storage at room temperature while poly(vinyl pyrrolidone) showed a slightly weak color intensity after 10 days (98.9% after 7days and 84.3% after 10 days). Therefore, these hydrophilic polymers can also be employed to stabilize H_2_O_2_ deposited on paper substrates.

## Conclusions

We mixed PVA with H_2_O_2_ before addition to the reaction wells of a PAD, and the degradation of H_2_O_2_ was suppressed. The method was quite simple and significantly improved the stability of the H_2_O_2_ deposited on a PAD. The H_2_O_2_ on a PAD had degraded in only one day without the addition of PVA at room temperature, whereas with the addition of 1 and 2% PVA, the H_2_O_2_ deposited on a PAD was stabilized for at least 30 days when stored at 4 °C in a refrigerator. Improving the stability of enzyme substrates for PADs is important for achieving point-of-care testing. Therefore, PVA is expected to be a useful stabilizer for other enzyme substrates. Even for use in developing countries where no refrigerator is available, the PADs can be transported with PVA under cooled conditions, although the PADs without PVA degrade immediately. Therefore, the PADs will work well in developing countries with poorly equipped laboratories when they are used immediately after transport. Other water-soluble polymers, such as poly(ethylene oxide), poly(ethylene glycol), and poly(vinyl pyrrolidone), would also improve the stability of enzyme substrates and other molecules such as enzymes, antibodies and nucleic acids. Thus, further investigation to explore a more effective stabilizer is expected to improve the practicality of PADs.

## Supplementary information

Long-term stabilization of hydrogen peroxide by poly(vinyl alcohol) on paper-based analytical devices

## Data Availability

All data generated or analysed during this study are included in this article (and its Supplementary Information files).
