# Supplementary material for: Long-term stabilization of hydrogen peroxide by poly(vinyl alcohol) on paper-based analytical devices
Source: Sci Rep. 2019 Sep 10;9:12951. doi: 10.1038/s41598-019-49393-6 (PMC6736875; doi:10.1038/s41598-019-49393-6)
Supplement: Supplementary file 1 — Long-term stabilization of hydrogen peroxide by poly(vinyl alcohol) on paper-based analytical devices [file 41598_2019_49393_MOESM1_ESM.pdf]

Supplementary Information

Long-term stabilization of hydrogen peroxide by poly(vinyl alcohol) on paper-based analytical devices

Tuchpongpuch Boonpoempoon[1], Wanida Wonsawat[1] & Takashi Kaneta[2*]

[1]Department of Chemistry, Faculty of Science and Technology, Suan Sunandha Rajabhat University, Bangkok, Thailand

[2]Department of Chemistry, Graduate School of Natural Science and Technology, Okayama University, Okayama, Japan

(a)

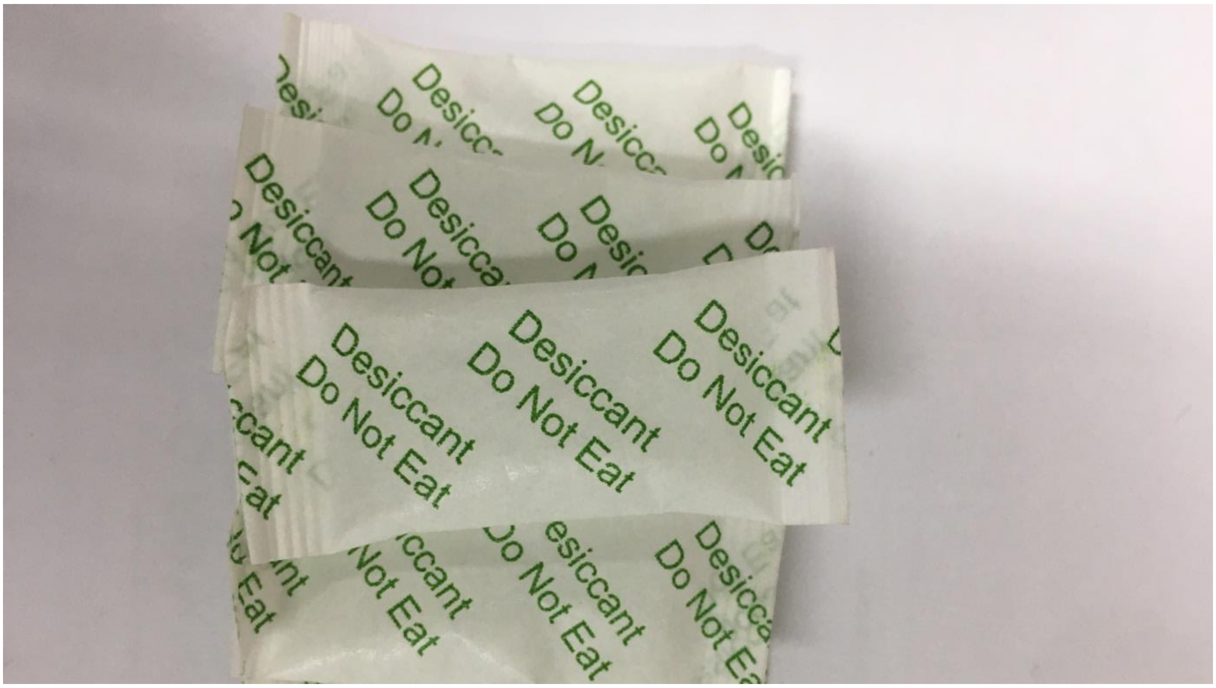

(b)

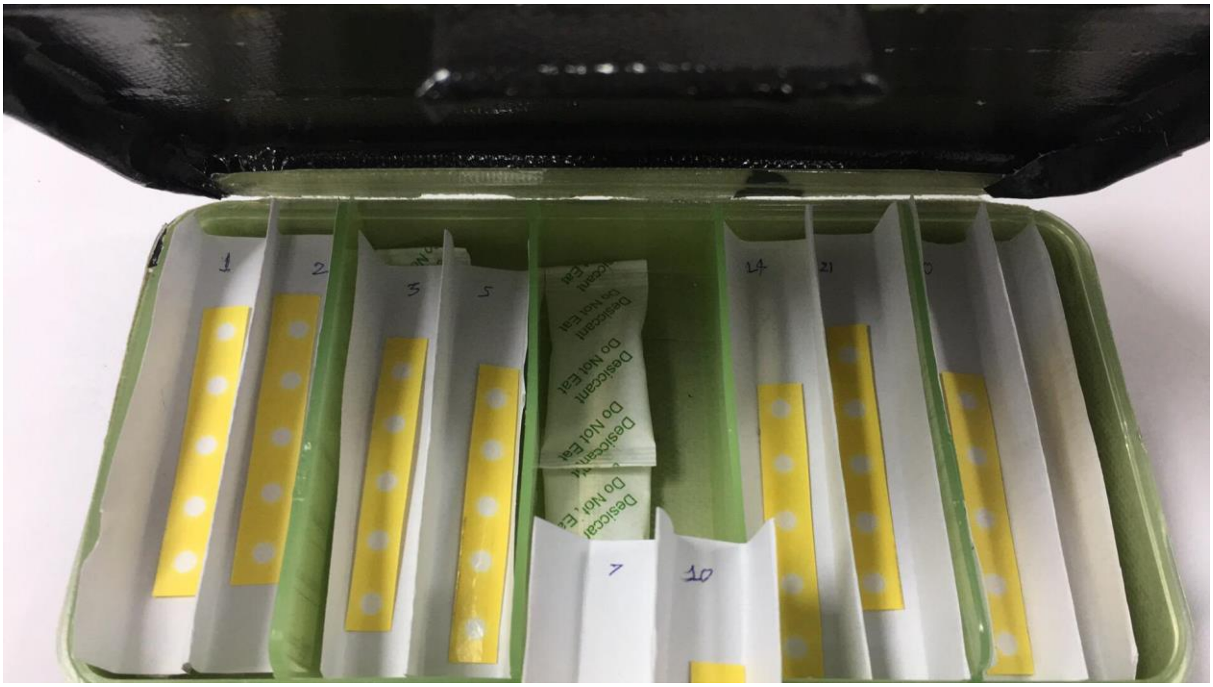

Fig. S1. Storage method for the PADs. (a) Silica gel and (b) PADs in the opaque box.

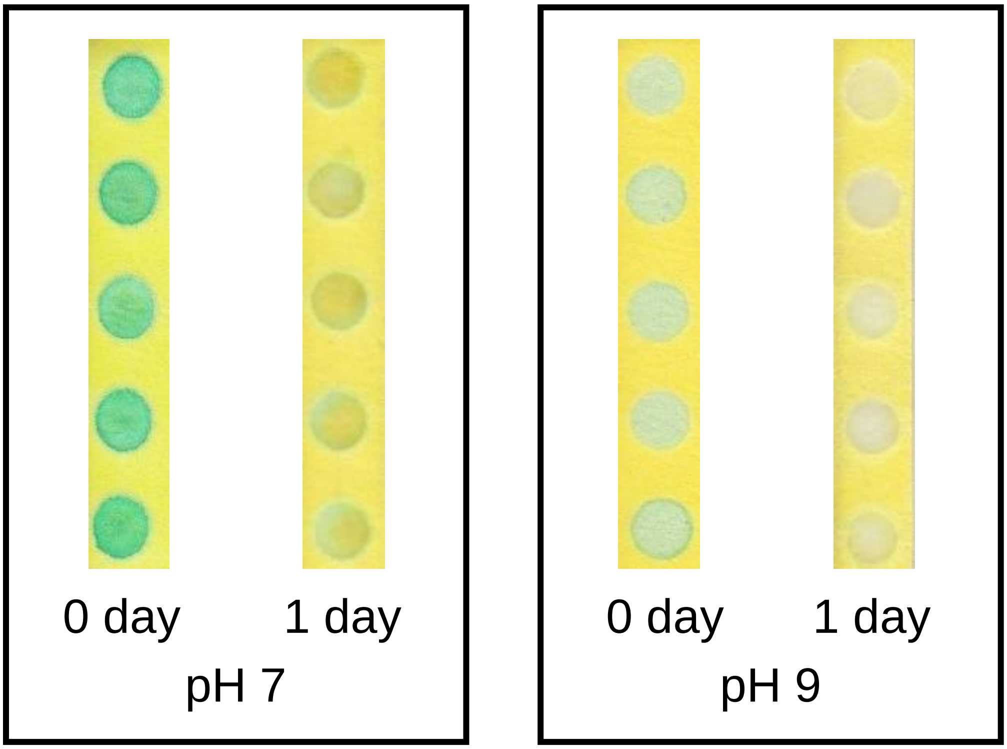

Fig. S2. Effect of pH on the storage of H_2O_2.
H_2O_2 solutions were prepared with 1 mM borate buffer (pH 9) and 1 mM phosphate buffer (pH 7) and stored at room temperature. The H_2O_2 solutions were added with HRP for the colorimetric reaction.

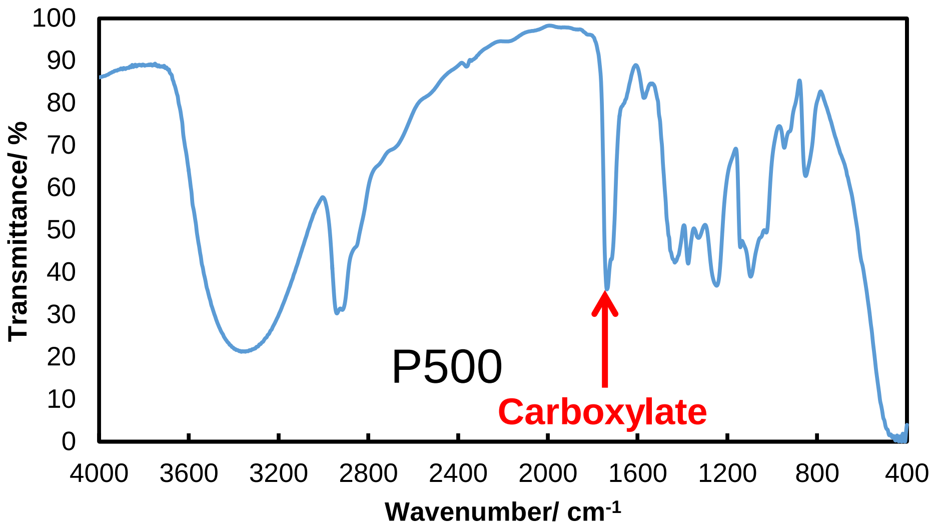

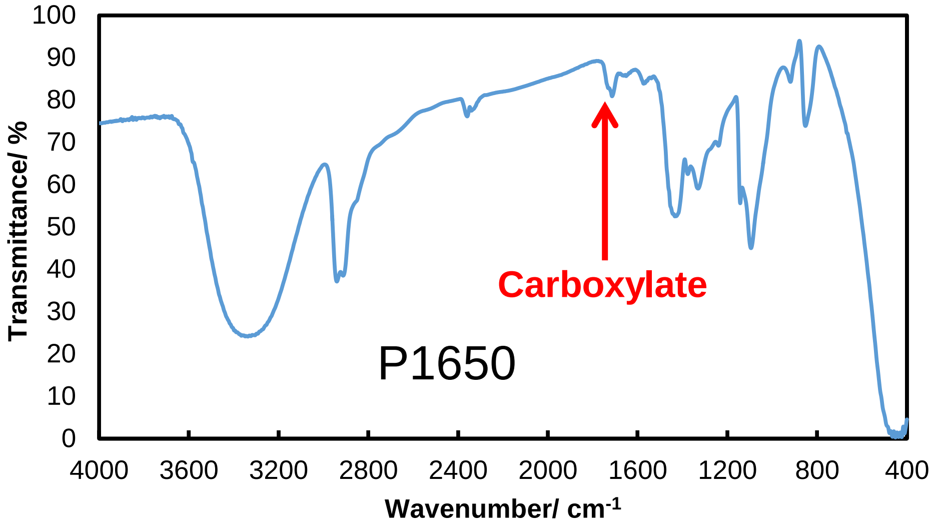

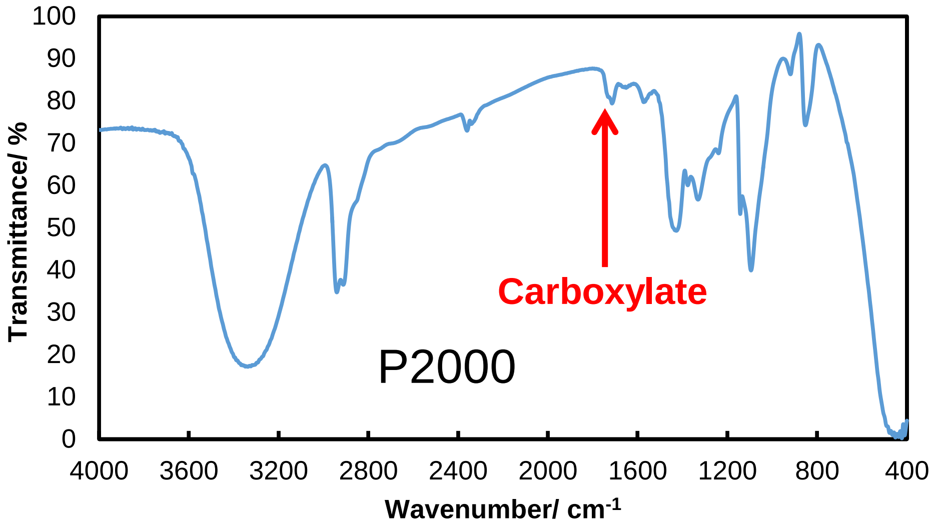

Fig. S3. Infrared spectra of PVAs.

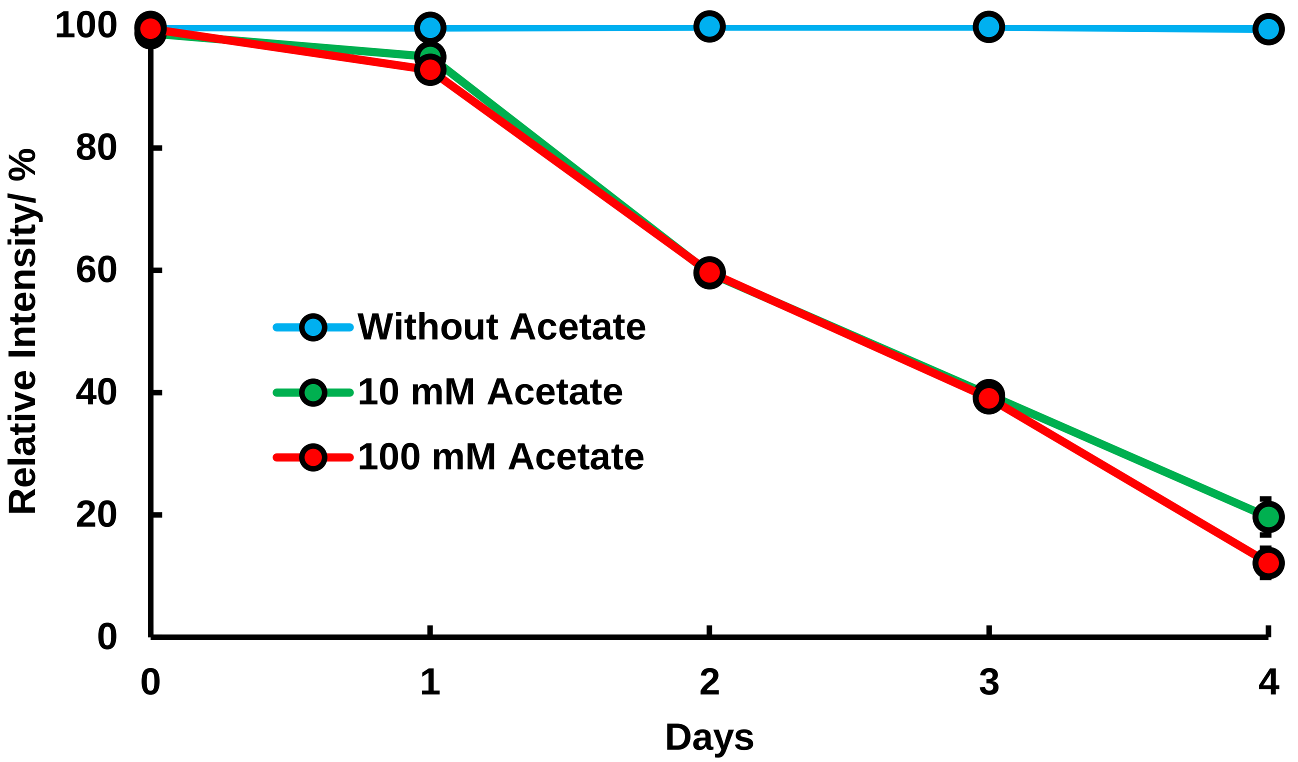

Fig. S4. Effect of sodium acetate.
A phosphate buffer without sodium acetate, with 10 mM sodium acetate, or with 100 mM sodium acetate was added to the PADs. The pHs of all buffers were adjusted at 6.5. The error bars indicate standard deviations.

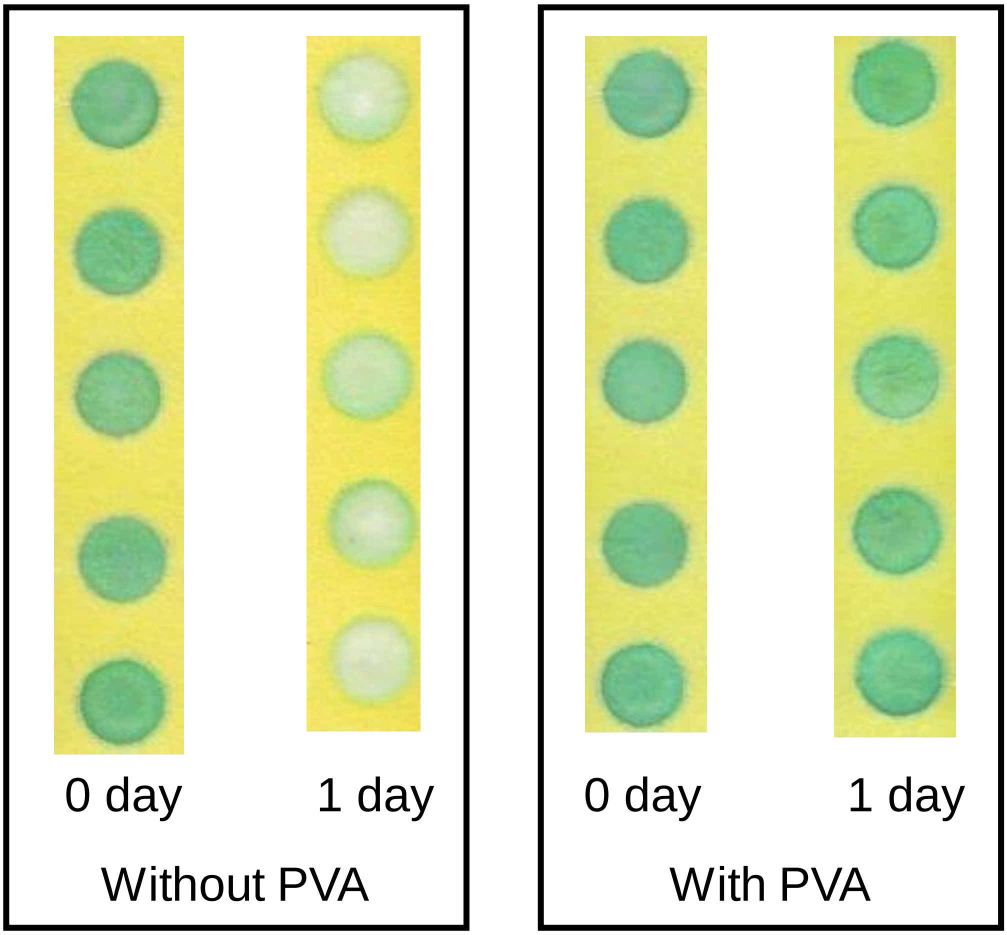

Fig. S5. Effect of Fe(III).

The reagents were added to the PADs as the following order; 5 µL of 35.7 mM TMB, 5 µL of 1 mg mL^{-1} BSA, 5 µL of 1 M phosphate buffer (pH6.5), 5 µL of 0.01% H_2O_2 with 2% PVA or without PVA, 5 µL of 100 µM $FeCl_3$. The results of 0 day were obtained immediately after drying the PADs. All PADs were stored in the refrigerator prior to the measurements after one day.
